# Supplementary material for: Chemical Targeting of the ATXN1 aa99–163 Interaction Site Suppresses polyQ-Expanded Protein Dimerization
Source: ACS Omega. 2025 Jun 17;10(25):27194–205. doi: 10.1021/acsomega.5c02465 (PMC12223882; doi:10.1021/acsomega.5c02465)
Supplement: Supplementary file 1 [file ao5c02465_si_001.pdf]

Supporting Information

Chemical targeting of the ATXN1 aa99-163 interaction site suppresses polyQ-expanded protein dimerization

Ioannis Gkekas[1,2,#], Katerina Pliatsika[1,2,#], Stelios Mylonas[3], Sotirios Katsamakas[4], Apostolos Axenopoulos[3], Simona Kostova[5], Erich E. Wanker[5], Dimitra Hadjipavlou-Litina[4], Konstantinos Xanthopoulos[1,2], Petros Daras[3] and Spyros Petrakis[1*]

[1] Institute of Applied Biosciences, Centre for Research and Technology Hellas, 57001 Thessaloniki, Greece

[2] Laboratory of Pharmacology, School of Pharmacy, Faculty of Health Sciences, 54124 Aristotle University of Thessaloniki, Thessaloniki, Greece

[3] Information Technologies Institute, Centre for Research and Technology Hellas, 57001 Thessaloniki, Greece

[4] Laboratory of Pharmaceutical Chemistry, School of Pharmacy, Faculty of Health Sciences, Aristotle University of Thessaloniki, 54124 Thessaloniki, Greece

[5] Max-Delbrueck-Center for Molecular Medicine in the Helmholtz Association, 13125 Berlin, Germany

[#] equal contribution

Table of contents

Supporting Tables S3
Supporting Figures S14

Supporting Tables

Table S1. Predicted interaction sites between ATXN1 and MED15 proteins along with the simulations in which these sites were identified.

Interaction site	Simulation
ATXN1 99-163	$ATXN1^{NT}$/MED15 (Model 1 & 2)
ATXN1 198-248	$ATXN1^{NT}$/MED15 (Model 1)
ATXN1 328-417	$ATXN1^{CT}$/MED15 (Model 1)
ATXN1 493-540	ATXN1(Q30)/MED15 (Model 2)
ATXN1 647-691	ATXN1(Q30)/MED15 (Model 1)
MED15 101-294	$ATXN1^{NT}$/MED15 (Model 1) & ATXN1(Q30)/MED15 (Model 1)
MED15 195-294	$ATXN1^{NT}$/MED15 (Model 1) & ATXN1(Q30)/MED15 (Model 1)
MED15 395-443	ATXN1(Q30)/MED15 (Model 2)
MED15 548-665	$ATXN1^{CT}$/MED15 (Model 1)
MED15 635-695	ATXN1(Q30)/MED15 (Model 1)

Table S2. Summary of MD simulation for the ATXN1-MED15 PPI. The table shows the time (in nanoseconds) of the MD simulation, the root mean square deviation (RMSD) of the protein-protein complex (in Å), the radius of gyration (Rg) (in Å) and the binding free energy (in kcal/mol) calculated over the simulation period.

Time (ns)	RMSD (Å)	Radius of Gyration (Rg) (Å)	Binding Free Energy (kcal/mol)
0	1.233390413	20.53983345	-10.24775633
1	1.167333387	20.63645966	-10.40618669
2	1.349256029	20.60584344	-10.19841672
3	1.170095652	20.61678289	-9.760286728
4	1.394108264	20.81145451	-9.876092542
5	1.32522846	20.62533	-10.22742317
6	1.288887956	20.56927624	-10.02419018
7	1.292328829	20.63225852	-10.10120192
8	1.285927571	20.70448506	-10.25072222
9	1.243923523	20.75320537	-10.15251764
10	1.161691807	20.46666841	-10.53448288
11	1.166870324	20.45511244	-10.95682898
12	1.188961143	20.22664954	-10.8453488
13	1.14023835	20.27762476	-11.00099787
14	1.13137454	20.19412859	-11.15719778
15	1.085961076	20.38834081	-10.73417704
16	1.066611404	20.3908357	-10.87524656
17	1.110124127	20.38352761	-11.02000647
18	1.144135442	20.26116532	-10.97272047
19	1.160860057	20.52709683	-10.60847443
20	1.155201131	20.45095285	-10.35121857
21	1.256572972	20.65988691	-10.6733866
22	1.215732922	20.67769528	-10.30404328
23	1.2835257	20.6773254	-10.39258958
24	1.323576293	20.6588815	-9.912523616
25	1.230009713	20.74204734	-9.905043432
26	1.255493396	20.65248874	-10.11242282
27	1.444115443	20.83178103	-10.08798086
28	1.308845568	20.55913411	-9.745234221
29	1.246280049	20.62931106	-10.37279783
30	1.236829172	20.34424823	-10.13531703
31	1.085426215	20.49489969	-10.56388336
32	1.062388493	20.3556512	-11.17491644
33	1.08948792	20.17308745	-11.21111501
34	1.121224795	20.41116159	-11.38842832
35	1.047757025	20.2793613	-10.89354861
36	1.126126869	20.34365811	-10.84542025

37	1.179740495	20.35384193	-10.8169487
38	1.113317333	20.39719901	-10.65332235
39	1.196239208	20.57058017	-10.41696394
40	1.087578267	20.54891044	-10.51817066
41	1.226851788	20.5648896	-10.40307103
42	1.329376684	20.75037584	-10.22719866
43	1.302676025	20.7087347	-9.944536836
44	1.245276346	20.70942462	-9.832297624
45	1.238210575	20.57639029	-9.871569657
46	1.337320445	20.66043841	-10.1095434
47	1.329840214	20.76271061	-10.31451467
48	1.272635771	20.74621218	-10.4112137
49	1.189725484	20.57299591	-10.4376036
50	1.257422176	20.60299783	-10.517525
51	1.152711133	20.39739186	-10.91269835
52	1.143744319	20.36069661	-10.68390103
53	1.114129559	20.26836731	-11.12946262
54	1.162605426	20.29878895	-11.01820792
55	1.108440314	20.29729279	-10.74278809
56	1.086740188	20.2900414	-10.80645183
57	1.265122183	20.33748974	-10.85313126
58	1.198582234	20.5687777	-10.62941442
59	1.126820106	20.29096278	-10.2816209
60	1.162059696	20.43393943	-10.25565869
61	1.314554235	20.54068851	-10.35980487
62	1.272492578	20.63822986	-10.13085468
63	1.173515418	20.79874609	-10.05905475
64	1.340104679	20.7330102	-10.29353595
65	1.306290487	20.56100723	-9.663966183
66	1.367331785	20.60783416	-10.19277046
67	1.282563227	20.65634159	-9.733883476
68	1.244534426	20.6355926	-10.20489378
69	1.257024968	20.51128054	-10.40452427
70	1.134037008	20.57024391	-10.59154464
71	1.231818063	20.45572659	-10.69574302
72	1.147359581	20.65673538	-10.87961025
73	1.122417677	20.49243393	-11.11207483
74	1.164872861	20.23529516	-10.92192414
75	1.081756435	20.25914327	-10.80926475
76	1.114411456	20.26874691	-10.38287692
77	1.140600422	20.35075308	-10.99664775
78	1.129657754	20.42522411	-10.44101827
79	1.219329076	20.48803316	-10.46985934

80	1.036228704	20.44654869	-10.64899419
81	1.291711058	20.61038072	-9.937401528
82	1.263220909	20.63198576	-10.08218102
83	1.298333824	20.57573131	-10.15360183
84	1.366074629	20.49811598	-10.0626218
85	1.208266544	20.50517436	-10.01281039
86	1.266115536	20.74489559	-10.47932158
87	1.255879782	20.70344547	-10.23938602
88	1.2708134	20.53134962	-10.28361045
89	1.149864478	20.55776191	-10.66644876
90	1.234720942	20.58681897	-10.23557236
91	1.203583458	20.40986473	-11.14980578
92	1.026966693	20.3581714	-10.96583487
93	1.180180648	20.25006208	-10.94919881
94	1.101495868	20.34322453	-10.82430066
95	1.08574593	20.42357565	-10.61826189
96	1.214489229	20.31650363	-11.15921909
97	1.093182606	20.28198517	-10.88471586
98	1.197612107	20.40301551	-10.68797643
99	1.095876053	20.49306131	-10.31052242
100	1.224798891	20.62646648	-10.49975704
**Median**	1.198582234	20.53134962	-10.4376036

Table S3. Aggregation-prone residues and their corresponding values. The table presents ATXN1 residues (aa90-170) along with their average aggregation-propensity values per amino acid (a4v) and the HSA (Hot-Spot Area) for each aa residue which define the aggregation profile (AP) of the polypeptide based on its complete sequence. Aminoacids were ranked according to their HSA value.

aa	a4v	HSA
H125	0.368	2.151
T126	0.214	2.151
F127	0.19	2.151
Q128	0.257	2.151
F129	0.02	2.151
I130	0.156	2.151
G131	0.223	2.151
S132	0.189	2.151
S133	0.015	2.151
Q134	0.232	2.151
Y135	0.069	2.151
G137	0.085	2.079
T138	0.277	2.079
Y139	0.274	2.079
A140	0.359	2.079
S141	0.142	2.079
F142	0.294	2.079
I143	0.508	2.079
V98	0.153	1.415
A99	0.18	1.415
T100	0.289	1.415
T101	0.421	1.415
L102	0.273	1.415
S145	0.356	1.241
Q146	0.345	1.241
L147	0.369	1.241
I148	0.091	1.241
V155	0.045	0.67
T156	0.049	0.67
S157	0.06	0.67
A158	0.06	0.67
V159	0.13	0.67
A160	0.157	0.67
S161	-0.002	0.67
A162	-0.002	0.67
A163	-0.006	0.67

V118	0.21	0.594
Q119	0.194	0.594
Y120	0.13	0.594
T151	0.082	0.349
A152	0.168	0.349
N153	0.039	0.349
H122	0.157	0.272
L123	0.075	0.272
A104	0.114	0.168
A105	0.005	0.168
Y106	-0.011	0.168
V115	-0.01	0.13
S116	0.099	0.13
R94	-0.004	0.095
S95	0.012	0.095
V96	0.027	0.095
S91	-0.204	0
A92	-0.067	0
P93	-0.028	0
P97	0.18	0
P103	0.289	0
A107	-0.045	0
T108	-0.185	0
P109	-0.185	0
Q110	-0.037	0
P111	-0.06	0
G112	-0.196	0
T113	-0.048	0
P114	-0.145	0
P117	0.036	0
A121	-0.029	0
P124	0.09	0
S136	-0.123	0
P144	0.492	0
P149	-0.105	0
P150	0.07	0
P154	0.018	0
G164	-0.03	0
A165	-0.286	0
T166	-0.396	0
T167	-0.396	0
P168	-0.504	0
S169	-0.376	0

Q170	-0.455	0

Table S4. Prediction and evaluation of binding pockets in the ATXN1^{NT} protein identified using the PockDrug online server. The table indicate volume hull, hydrophobic kyte, polar and aromatic residues proportion, frequency of Otyr atoms, number of pocket residues, druggability probability score and standard deviation.

Pocket	Volume Hull	Hydrophobic kyte	Polar residues proportion	Aromatic residues proportion	Otyr atom	Number of pocket residues	Druggability Probability	STDEV
**1**	1193.85	-0.05	0.4	0.13	0.02	15	0.81	0.04
**2**	796.71	-0.34	0.56	0.25	0	16	0.74	0.1
**3**	3436.6	-0.43	0.49	0.14	0.01	35	0.71	0.02

Table S5. Binding score of the selected and AI-predicted compounds (n=24) with high affinity for ATXN1 aa99-163.

ChemBridge code	IUPAC NAME	Score
6418951	2-{[4-(3,4-dihydro-2(1H)-isoquinolinylmethyl)benzoyl]amino}benzoic acid	0.979
5466832	3-(aminocarbonyl)-1-[2-(4-biphenylyl)-1-methyl-2-oxoethyl]pyridinium bromide	0.974
6015799	ethyl 4-{5-[({3-[(2-methylbenzoyl)amino]phenyl}amino)carbonyl]-2-furyl}benzoate	0.975
5759625	2-[3-(1,3-benzoxazol-2-yl)phenyl]-1,3-dioxo-5-isoindolinecarboxylic acid	0.989
5922064	3-[(3-{[(4-phenoxyphenyl)amino]sulfonyl}benzoyl)amino]benzoic acid	0.968
5871031	4-(benzyloxy)-N-(3-[1,3]oxazolo[4,5-b]pyridin-2-ylphenyl)benzamide	0.982
573393	1,3-dioxo-2-[4-(5-phenyl-1,3,4-oxadiazol-2-yl)phenyl]-5-isoindolinecarboxylic acid	0.975
7629383	N-3-quinolinyl-9H-fluorene-2-sulfonamide	0.989
7779825	(4-{[(2-benzyl-1,3-dioxo-2,3-dihydro-1H-isoindol-5-yl)carbonyl]amino}phenoxy)acetic acid	0.975
7933413	N-[3-(2-quinoxalinyl)phenyl]benzamide	0.984
6433773	5-[(2-methylbenzoyl)amino]-N-(3-methylphenyl)-1-phenyl-1H-pyrazole-4-carboxamide	0.976
5751467	N-(2-methyl-5-[1,3]oxazolo[4,5-b]pyridin-2-ylphenyl)-2-naphthamide	0.972
5160674	2-dibenzo[b,d]furan-3-yl-1,3-dioxo-5-isoindolinecarboxylic acid	0.986
6238693	3'-[(9H-fluoren-2-ylcarbonyl)amino]-4-biphenylyl acetate	0.99
6048589	4-{4-[(diphenylacetyl)amino]benzoyl}phthalic acid	0.993
6052953	2-({[4-(2-naphthyloxy)phenyl]amino}carbonyl)benzoic acid	0.984
7969487	N-{3-[(1,3-dioxo-2,3-dihydro-1H-isoindol-5-yl)oxy]phenyl}-2-phenylbutanamide	0.923
6846586	4-tert-butyl-N-[3-(5-phenyl-1,3,4-oxadiazol-2-yl)phenyl]benzamide	0.985
5561416	1-(4-biphenylyl)-2-{2-imino-3-[2-(4-morpholinyl)ethyl]-2,3-dihydro-1H-benzimidazol-1-yl}ethanone hydrobromide	0.964
5606311	N-1-naphthyl-N'-{3-[2-(2-pyridinyl)ethyl]phenyl}urea	0.979
5319422	3-(benzoylamino)-1-[2-(9H-fluoren-2-yl)-2-oxoethyl]pyridinium bromide	0.981
6032246	3-(benzyloxy)-N-(2-methyl-3-[1,3]oxazolo[4,5-b]pyridin-2-ylphenyl)benzamide	0.958
5780637	cyclohexyl (4-methyl-3-{[(1-naphthylamino)carbonyl]amino}phenyl)carbamate	0.988
5755483	2'-({[4-(3-oxo-3-phenyl-1-propen-1-yl)phenyl]amino}carbonyl)-2-biphenylcarboxylic acid	0.967

Table S6. Key residues between ATXN1 aa99-163 and compound 5755483 identified through molecular docking and MD simulations. The table lists the interacting residues, the interaction type, the frequency (%) of the interaction during the simulation, the mean interaction distance (nm) and the binding free energy (kcal/mol).

Residue	Interaction Type	Interaction Frequency (%)	Mean Distance (nm)	Binding free energy (kcal/mol)
Thr^{113}	Hydrophobic	65	0.2	-2.5
Pro^{114}	Hydrophobic	65	0.24	-2.2
Tyr^{135}	Pi-Pi stacking	67	0.35	-4.1
Ser^{136}	Hydrogen bond	85	0.3	-5.8

Table S7. Primer sequences used for the generation of deletion-carrying ATXN1 and MED15 Gateway entry clones.

Deletion clone	Forward Primer (5'-3')	Reverse Primer (5'-3')
ATXN1 Δ99-163	GGGGCCACCACTCCATC	CACGGGGACAGACCTGGG
ATXN1 Δ198-248	CAGTACGTCCACATTTCCAGTTCT	CTGCTCAGCCTTGTGTCCCGG
ATXN1 Δ328-417	GGCCTGCATTTAGGGAAGC	CAGGACCTCCTTGGCCTG
ATXN1 Δ647-691	AACCTGAAGAACGGCTCTGTT	TTCAACGCTGACCTGGGC
ATXN1 Δ493-540	CTGGTCACCCAGGCCGCC	GGGCTGTGTGCCGGGGATC
MED15 Δ101-294	CACACACAGCACCACCAG	GCCAATTCCAGCGGCTCC
MED15 Δ635-695	TGGATAGACCGGCAGTGGCAG	GATGCTCTGCCGCTCATCATC
MED15 ΔCC	CCATCACAACTCCCGCCACAGTCGC	TCCCGCAGCAGGTCCGCCAGTCAGG
MED15 Δ195-294	CACACACAGCACCACCAG	ACTCTGCTGAGCCTGGAACT
MED15 Δ395-443	CCCTCACCGCAGCCCTCC	CGAGGACAGCATGGGGAGG
MED15 Δ548-665	CTGATCTGCAAGCTGGATGACA	CTTTTGCAAGGTCTTCAGGGG
ATXN1 ΔAXH	CTTACCCTCAAGAACCTGAAGAACG	CACAGGCAGGTGGATCTGGGCCTGCA

Supporting Figures

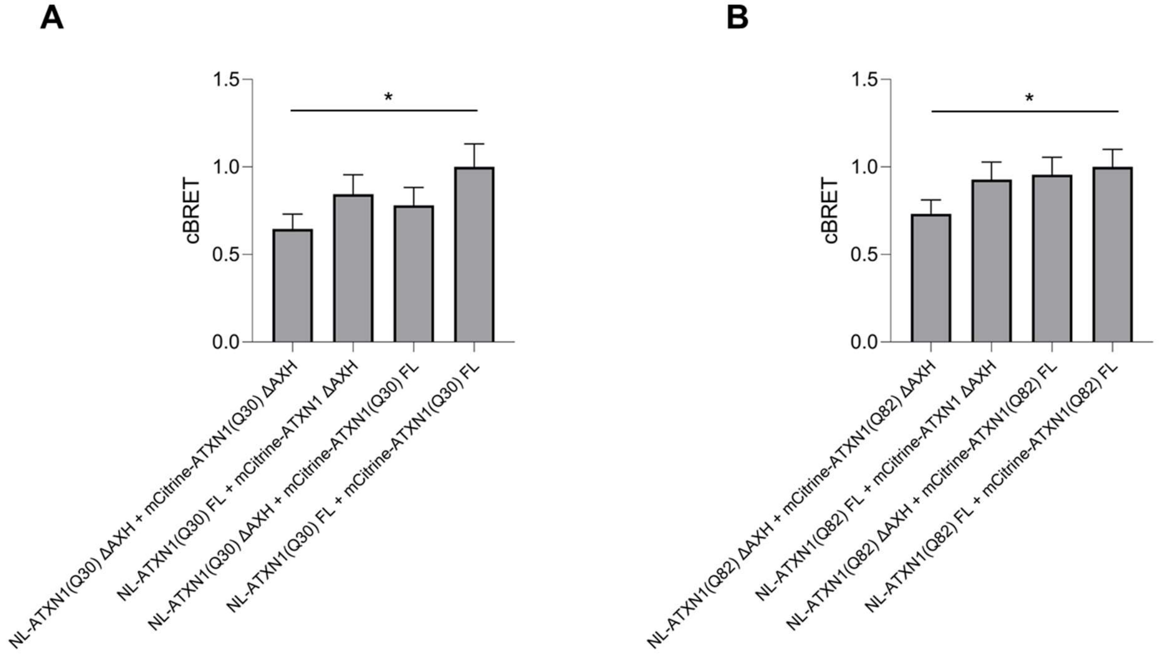

Figure S1. Quantification of **(A)** wild-type (Q30) and **(B)** polyQ-expanded (Q82) ATXN1 homo-dimerization harboring an AXH domain deletion. Deletion of the AXH domain (ΔAXH) moderately reduces ATXN1(Q30) or (Q82) dimerization compared to the respective full-length (FL) proteins. Data are presented as mean ± standard deviation (SD).

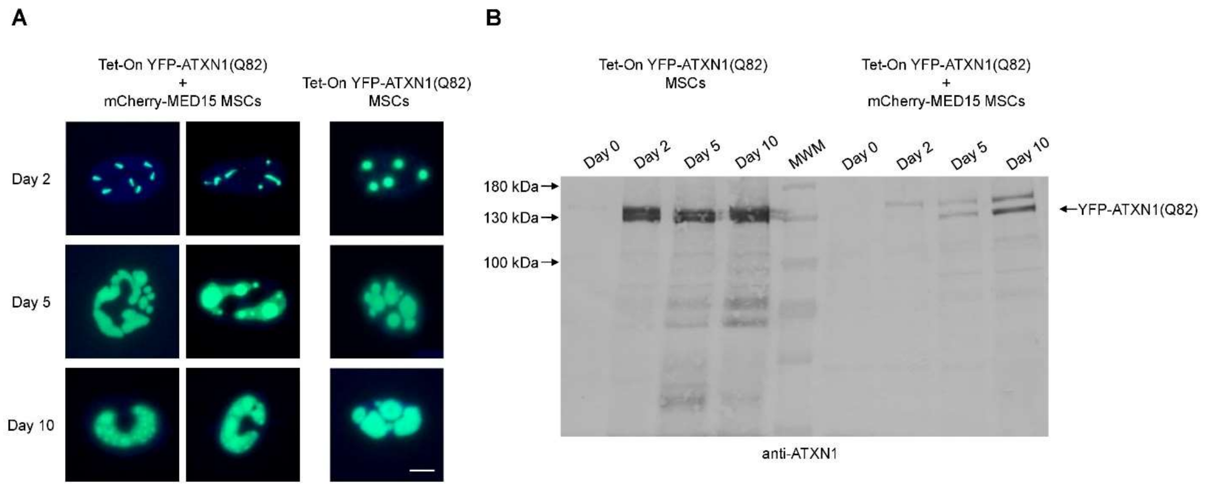

Figure S2. (A) Micrographs of representative YFP-ATXN1(Q82) IIBs at different time points in the presence (left panels) or absence (right panel) of mCherry-MED15 protein. Scale bar: 10 μM. **(B)** Uncropped Western blot of Figure 1E for YFP-ATXN1(Q82).

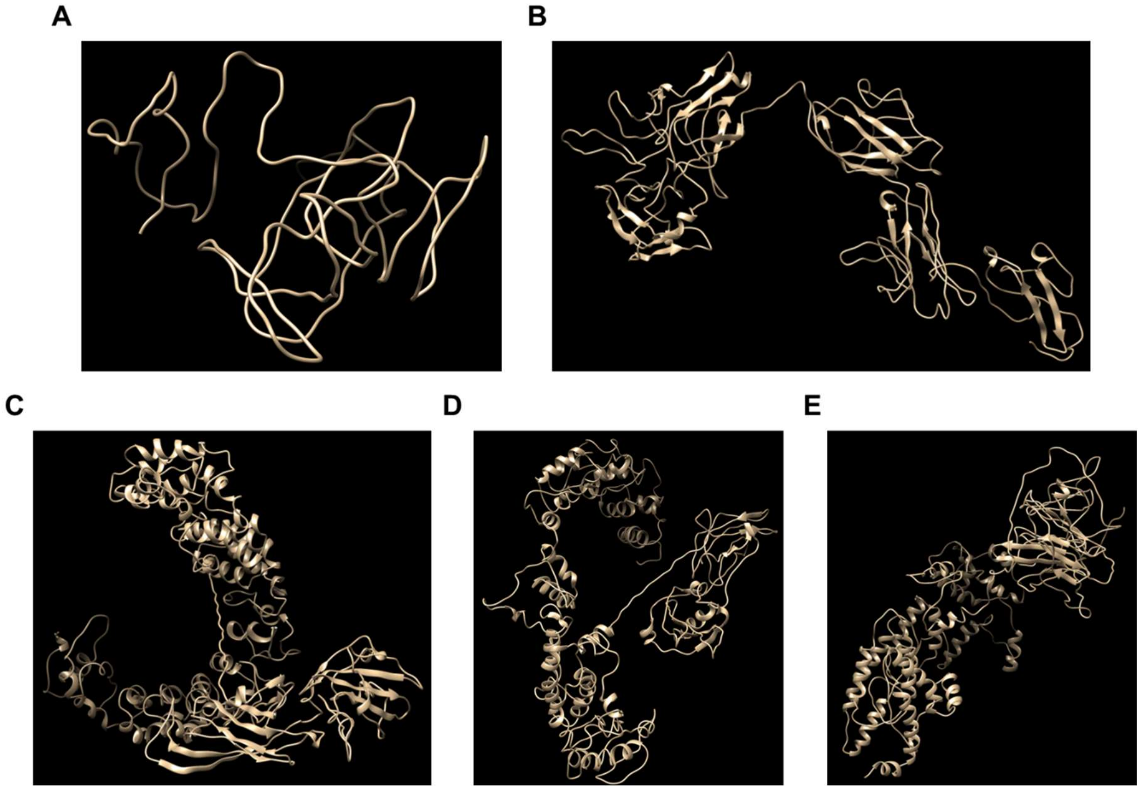

Figure S3. Predicted structures of ATXN1 or MED15 proteins using the I-TASSER software. **(A)** ATXN1^{NT}, **(B)** ATXN1^{CT}, **(C)** ATXN1(Q30), **(D)** MED15 (model 1) and **(E)** MED15 (model 2)

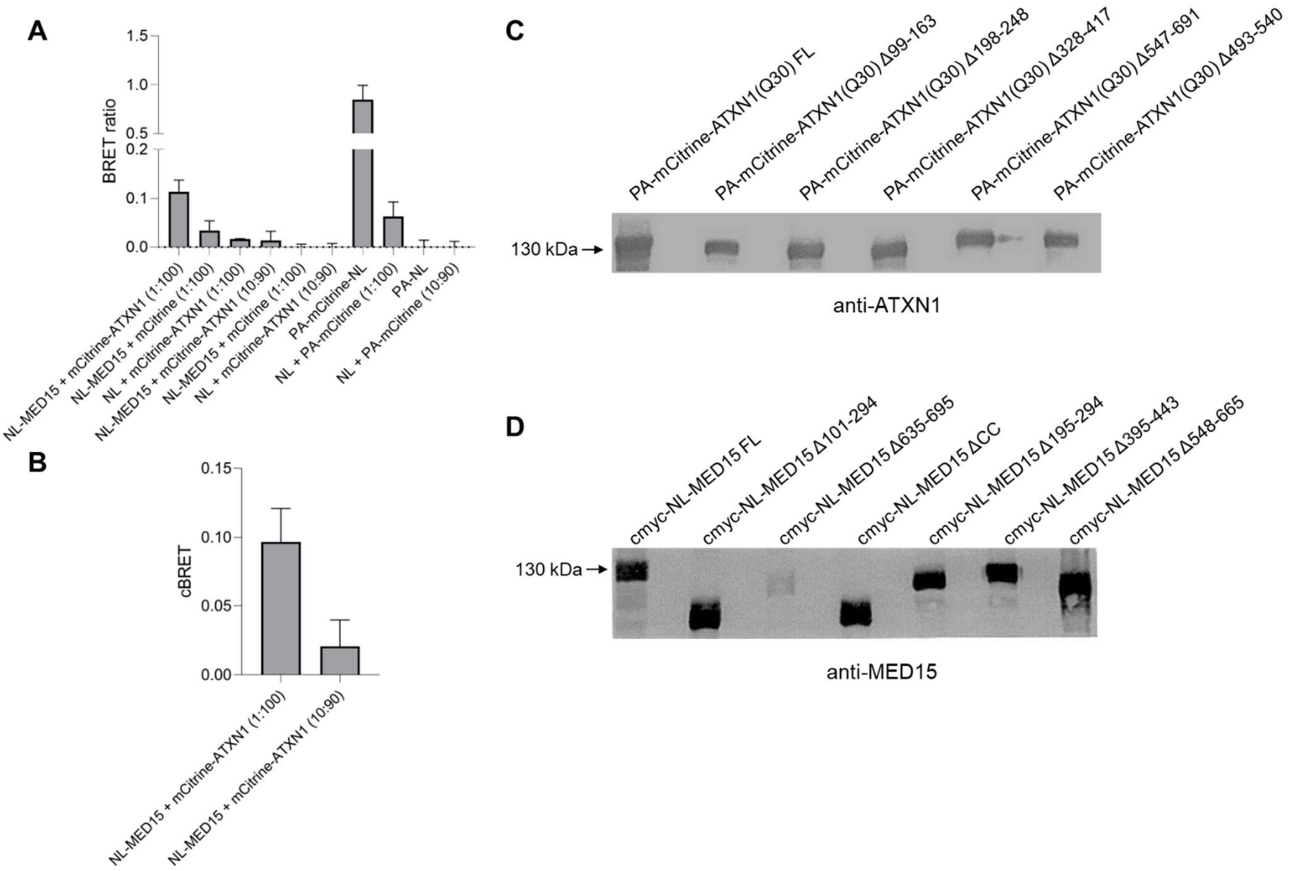

Figure S4. Quantification of ATXN1-MED15 PPI using LuTHy assay. **(A)** BRET ratios for ATXN1-MED15 PPI. The proteins PA-mCitrine-ATXN1 and NL-MED15 (at a 1:100 ratio), along with the positive control PA-mCitrine-NL, show significantly elevated BRET ratios, indicating a strong interaction. **(B)** cBRET ratios confirming the efficient quantification of ATXN1-MED15 PPI at a 1:100 ratio of PA-mCitrine-ATXN1/NL-MED15. **(C)** Immunoblot for ATXN1 in extracts from transiently transfected HEK293T producing full-length or deletion-carrying ATXN1 protein. **(D)** Immunoblot for MED15 in extracts from transiently transfected HEK293T producing full-length or deletion-carrying MED protein.

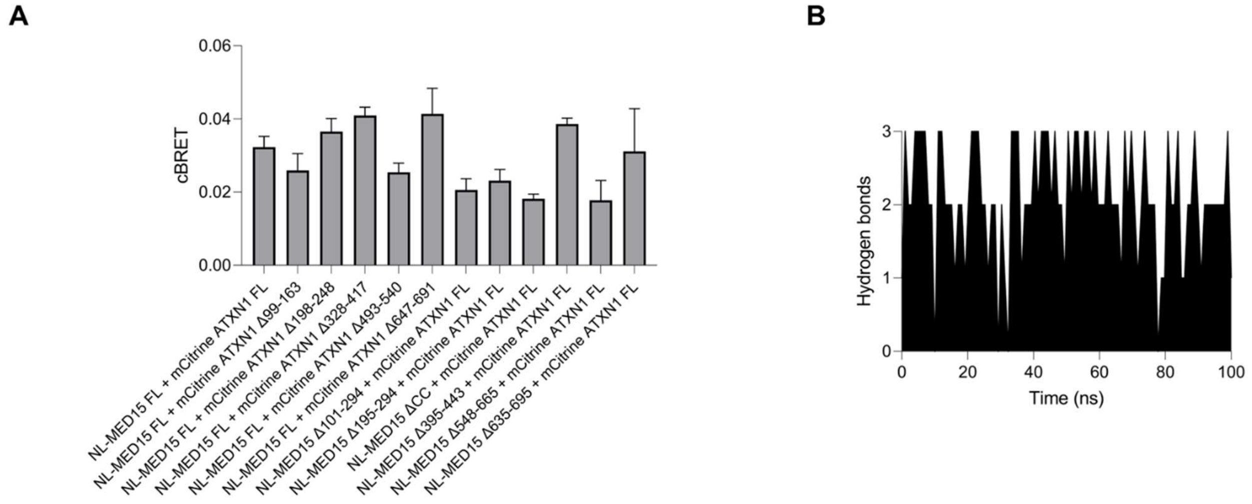

Figure S5. (A) Quantification (cBRET ratio) of the interaction between full-length and deletion-carrying ATXN1/MED15 proteins. Results were compared to the interaction of full-length ATXN1 and MED15. **(B)** The number of hydrogen bonds formed between domains 99-163 of ATXN1 and 548-665 of MED15 over a 100 ns simulation.

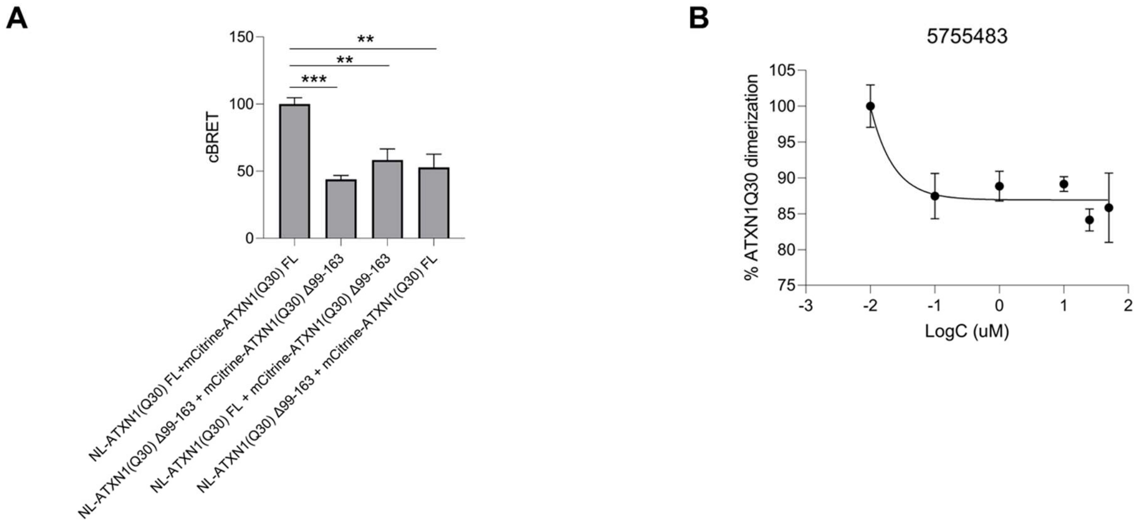

Figure S6. The aa99-163 domain mediates the homo-dimerization of wild-type ATXN1 in cell models. **(A)** Quantification of the homo-dimerization of full-length or Δ99-163 ATXN1(Q30) using the LuTHy assay. The bar graph shows various combinations of full-length or deletion-carrying ATXN1 proteins. Data in bar graphs are shown as mean ± SD. ** p-value <0.01, *** p-value < 0.001). **(B)** Dose-dependent effect of compound 5755483 in the homo-dimerization of ATXN1(Q30).

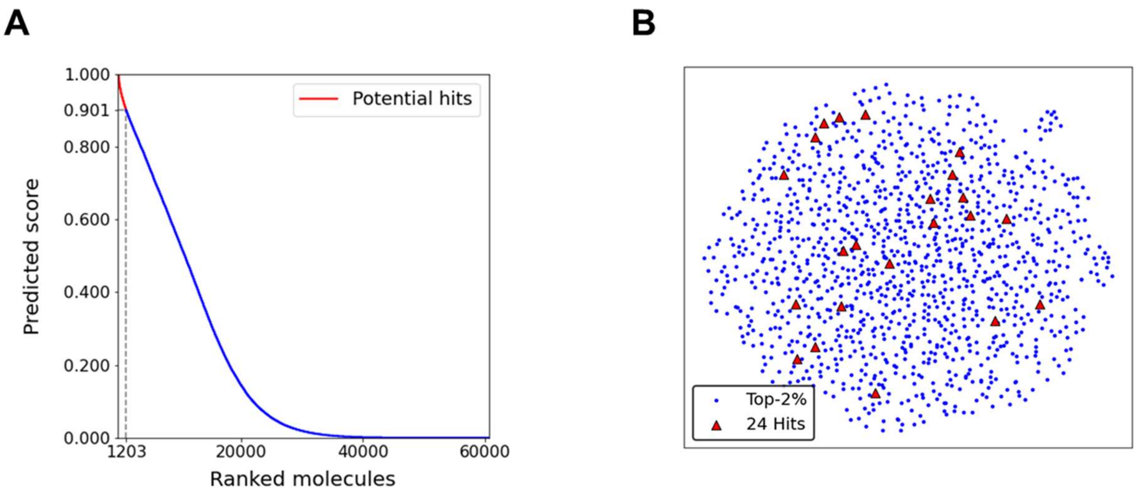

Figure S7. (A) Molecule ranking scores after applying the virtual screening pipeline to the initially filtered library of approximately 60,000 molecules, followed by the selection of the top 2% (1,203 molecules). **(B)** UMAP visualization of the final selected hits (n=24) compared to the top 2% of screened molecules, projected onto a reduced feature space defined by shape descriptors. This visualization highlights the distribution and clustering of the final hits relative to the broader set of top-ranked molecules.

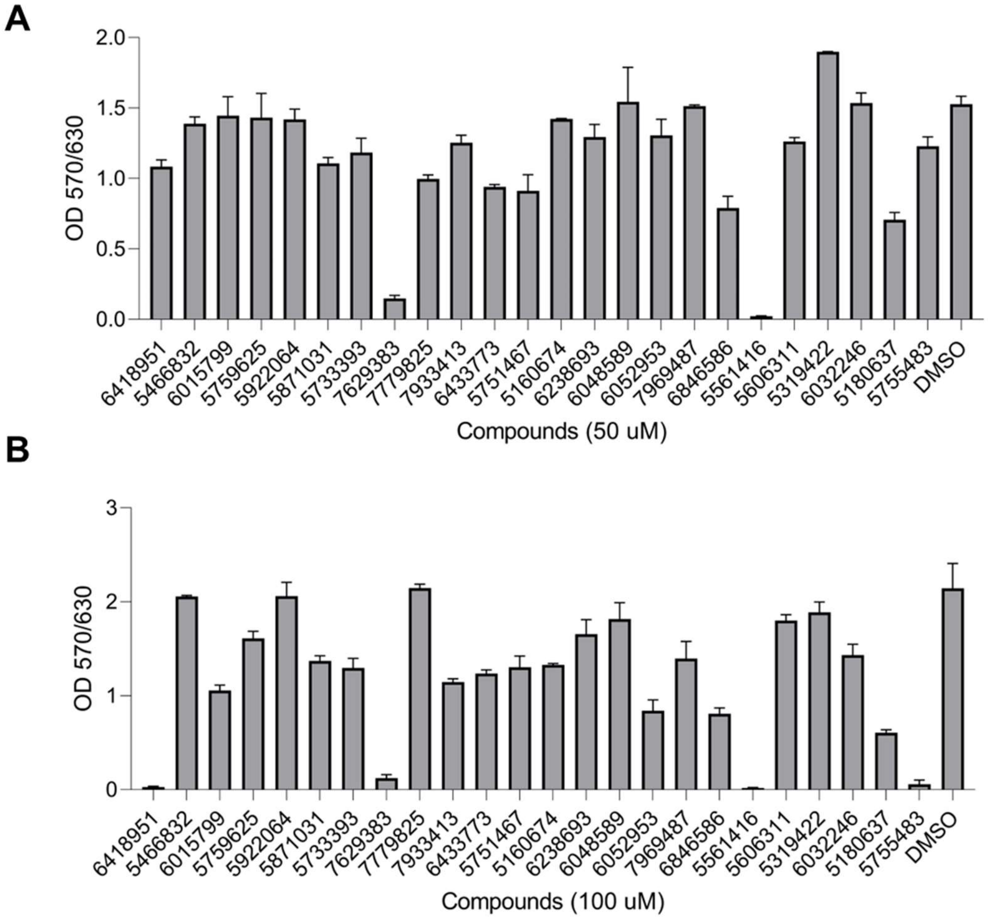

Figure S8. Viability of HEK293T cells following a 48-hour treatment with compounds at concentrations of **(A)** 100 μM and **(B)** 50 μM, compared to control treatment with DMSO.

6032246

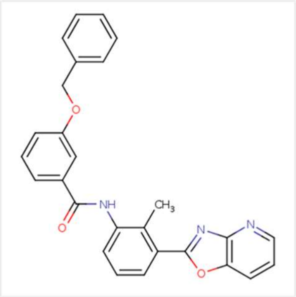

6846586

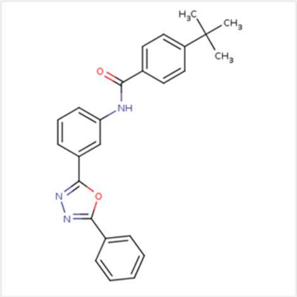

5751467

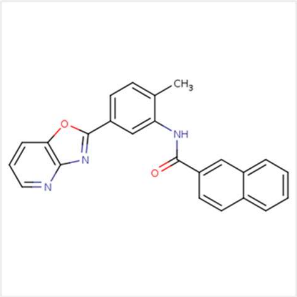

5319422

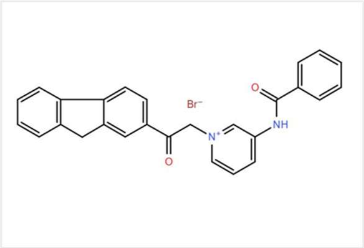

7969487

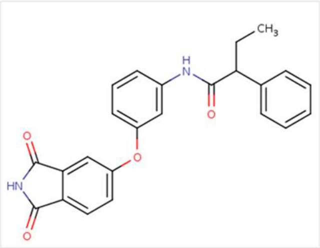

Figure S9. Chemical structures of five compounds (Chembridge ID: 6032246, 6846586, 5751467, 5319422, 7969487) reducing ATXN1-MED15 PPI.

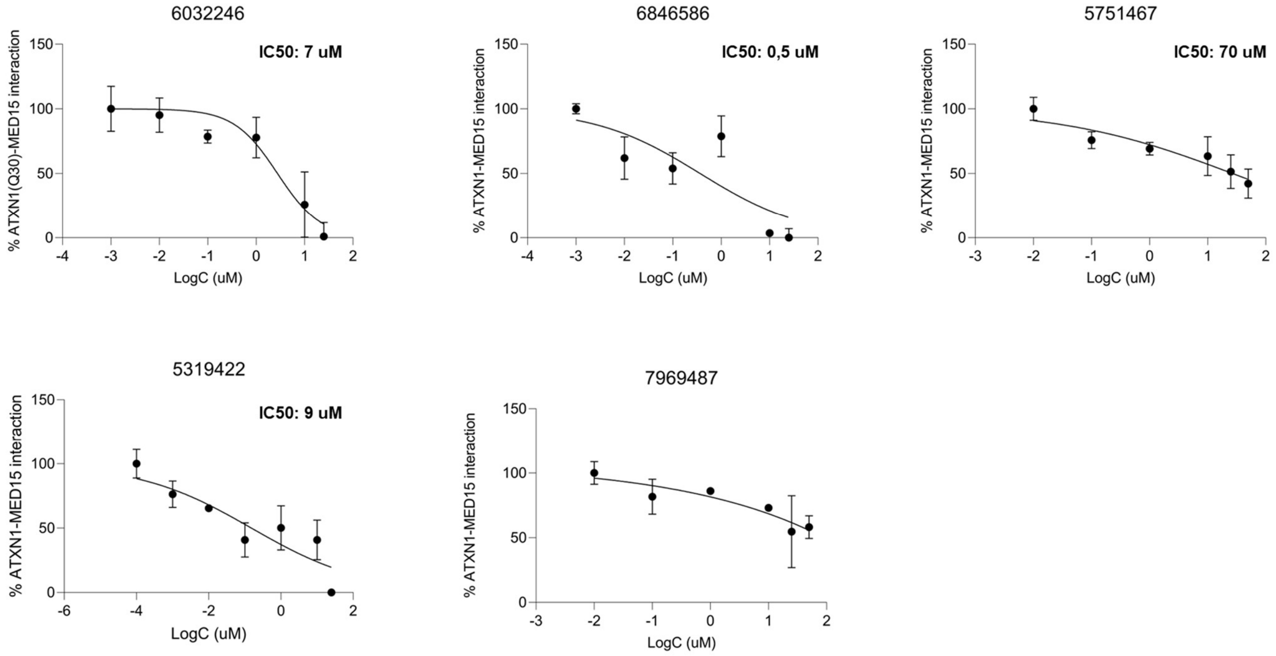

Figure S10. Concentration-response curves for the inhibitory effect of selected compounds on ATXN1-MED15 PPI.

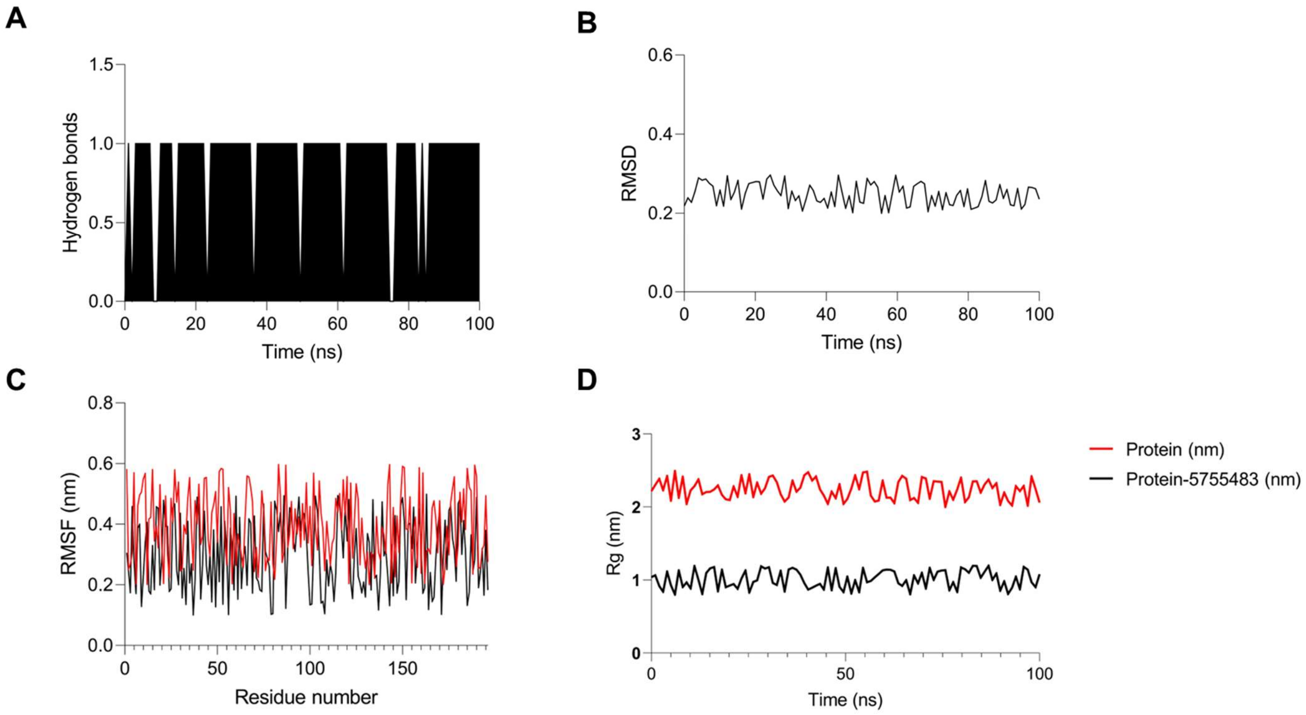

Figure S11. MD simulation for the interaction between ATXN1 aa99-163 and compound 5755483. **(A)** The number of hydrogen bonds formed between ATXN1 and 5755483 over 100 ns of simulation. **(B)** RMSD of the ATXN1-5755483 complex over the simulation time. **(C)** RMSF and **(D)** Radius of gyration (Rg) of the ATXN1 protein in the presence (black) or absence (red) of compound 5755483. The lower Rg values of the bound state indicate that compound 5755483 promotes a more stable protein conformation.
